# Epidemiology, Risk Factors, and Predictors of Disability in a Cohort of Jordanian Patients with the First Ischemic Stroke

**DOI:** 10.1155/2020/1920583

**Published:** 2020-06-04

**Authors:** Majdi Al Qawasmeh, Belal Aldabbour, Aiman Momani, Deema Obiedat, Kefah Alhayek, Raid Kofahi, Ahmed Yassin, Khalid El-Salem

**Affiliations:** Department of Neurology, King Abdullah University Hospital (KAUH) of Jordan University of Science and Technology (JUST), Irbid, Jordan

## Abstract

**Objective:**

To identify the risk factors, etiologies, length of stay, severity, and predictors of disability among patients with the first ischemic stroke in Jordan.

**Methods:**

A retrospective cohort study of 142 patients who were admitted to the Neurology Department at King Abdullah University Hospital between July/2017 and March/2018 with a first ischemic stroke. Etiology was classified according to the TOAST criteria. Severity was represented by NIHSS score, disability by mRS score, and prolonged length of stay as hospitalizations more than 75^th^ percentile of the cohort's median length of stay. Analysis of the sample demographics and descriptive statistics were done, including frequencies of prevalence of independent variables (risk factors) and frequencies of stroke and etiology work-up. Chi-square and univariate analysis of variance “ANOVA” were used to investigate the relationship between risk factors and type of stroke. Finally, logistic regression analysis was used to measure the contribution of each of the independent variables. IRB approval was obtained as necessary.

**Results:**

The mean age for the cohort was 66.5 years. The most common risk factors were hypertension (78.8%), diabetes mellitus (60.5%), and ischemic heart disease (29.4%). The most common stroke etiology was small-vessel occlusion (54.2%). Median length of stay was 4 days. Prolonged length of stay was observed in 23.23% of patients, which was associated with several factors, the most common of which were persistent dysphagia (57.5%), nosocomial infection (39.3%), and combined dysphagia and nosocomial infection (21.2%). The mean admission NIHSS score was 7.94, and on discharge was 5.76. In-hospital mortality was 2.81%, while 50% of patients had a favorable outcome on discharge (mRS score between 0-2). The mean discharge mRS score for the cohort was 2.47 (SD ± 1.79). Large artery atherosclerosis was associated with the highest residual disability with a mean score of 3.67 (SD ± 1.88), while the stroke of undetermined etiology was associated with the lowest residual disability with a mean score of 1.60 (SD ± 1.78). Significant predictors of mRS score were smoking (*t* 3.24, *P* < 0.001), age (*t* 1.98, *P* < 0.049), and NIHSS score (*t* 9.979, *P* 0.000).

**Conclusion:**

Ischemic strokes have different etiologies that are associated with different levels of impact on the patient's clinical status and prognosis. Large artery atherosclerosis was associated with the highest residual disability. Regarding predictors of prognosis, current smoking status, age above 50, gender, and NIHSS on admission appear to be the strongest predictors of prognosis. Finally, higher NIHSS score on admission resulted in a longer hospital stay.

## 1. Introduction

Stroke is an important cause of mortality and morbidity worldwide. Recent studies recognize this disease to be the second leading cause of death and 3^rd^ major cause of disability globally [1, 2]. Despite a decline in stroke incidence in high-income countries, the global burden of stroke is increasing. This trend is largely attributable to an increase in stroke incidence and prevalence as well as stroke-related deaths and disability in low and middle-income countries, reflecting suboptimal preventive strategies [3, 4]. Stroke risk factors include nonmodifiable factors such as age, sex, and race, as well as modifiable ones like hypertension, smoking, alcohol, obesity, physical inactivity, diabetes mellitus, hyperlipidemia, and atrial fibrillation [5, 6].

In Jordan, stroke is believed to be the 2^nd^ leading noncommunicable cause of death in the country, tracking closely behind ischemic heart disease [7]. In a study in 2004, which evaluated 200 patients with ischemic stroke in Jordan University Hospital “JUH”, the mean age of patients was 61.2 years, while the most common stroke etiology identified was lacunar infarct (51.5%). Hypertension, diabetes mellitus, and smoking were the most common risk factors for atherosclerotic noncardioembolic strokes identified by that study [8]. A more recent study in 2015 evaluated ischemic stroke etiologies and risk factors in 100 patients, who were observed at JUH over a one-year-period. In this study, the mean age was 66 years, and lacunar stroke remained the most common stroke etiology identified (36%). The most common risk factor was hypertension, followed by hyperlipidemia, and diabetes mellitus [9].

However, there are no published studies that have measured stroke severity or poststroke disability among patients with ischemic stroke in Jordan. Measuring stroke severity and stroke-related disability is very important for weighing the economic burden of this disease and also for efforts to design evidence-based preventive and rehabilitation strategies.

## 2. Methodology

### 2.1. Study Design and Population

This is a retrospective cohort study that was conducted at King Abdullah University Hospital “KAUH”, the largest tertiary teaching hospital located in the north of Jordan. KAUH has over 680 beds. It offers outpatient and inpatient health care services to residents of all provinces in the northern part of the country, who constitute over 25% of the total population of the country [10]. It, also, receives referrals from other hospitals in the north of Jordan. Ethical approval for this study was granted by the institutional review board committee at Jordan University of Science and Technology “JUST”.

### 2.2. Study Population and Case Identification

The study population consisted of patients above the age of sixteen who were admitted to the Neurology Department at KAUH between July 1^st^, 2017, and March 31^st^, 2018, with the primary diagnosis of first-time ischemic stroke. The study used secondary data that were extracted from the hospital's electronic medical record system.

### 2.3. Data Collection

Data for all patients who were admitted to KAUH with stroke as the primary diagnosis were extracted. After that, data on patients who did not meet the inclusion criteria for the study were excluded, which consisted of patients with a final diagnosis other than an ischemic stroke (e.g., hemorrhagic strokes, transient ischemic attacks, or others), patients with previous strokes, and patients in whom the ischemic stroke occurred in the setting of another major acute illness. All records with missing essential data fields were excluded. Finally, data for patients in the study sample (patients who met the inclusion criteria) were purged of all unnecessary fields. Retained data fields included age, sex, length of stay “LOS”, reason for prolonged hospitalization, NIHSS scores on admission and discharge, mRS score on discharge, and relevant risk factors (hypertension, diabetes mellitus, hyperlipidemia, smoking status, ischemic heart disease, atrial fibrillation, and heart failure). Thrombophilia and/or vasculitis test results—if performed—were also retained. Radiology images were reviewed by the research team, and data were also collected from the database and were compared with the official radiology reports. The imaging modalities used and the findings on brain imaging and brain and neck angiography were all documented.

### 2.4. Key Measures

For the purpose of this study, ischemic stroke was defined according to the 2013 AHA definition [11]. Etiology was identified according to TOAST criteria [12]. Stroke due to large artery atherosclerosis was determined in cases associated with proximal intracranial or extracranial atherosclerosis causing luminal stenosis (≥50%) or occlusion in arteries that correspond to the ischemic area [12]. Cardioembolic stroke was defined as cases where a definite cardiac source of embolism was documented in the absence of significant ipsilateral large vessel disease [12, 13]. Lacunar stroke was defined as a noncortical stroke smaller than 16 mm without a cardioembolic source or significant large vessel stenosis [12, 13]. Stroke in the young was defined as ischemic stroke occurring in patients 50 years of age or younger [14]. Hypertension, diabetes mellitus, and hyperlipidemia were defined according to standard guidelines [15–17] or if the patient has been on drug treatment for these conditions. Atrial fibrillation “AF” was ascertained from ECG on admission or on previous visits and from Holter monitoring reports. Ischemic heart disease and heart failure were identified based on cardiologist notes from previous visits, history of cardiac catheterization with stenting, or from recent consultation and/or echocardiography findings. Severity and disability scores were documented using admission and discharge NIHSS and mRS scores, respectively. Several cutoff points for stroke severity exist in the literature. Our institution's local protocol was used, which divides ischemic stroke into mild (NIHSS less than 6), moderate (NIHSS 6-15), severe (16–25), and very severe (more than 25). Favorable stroke outcome was defined as mRS scores ≤ 2, and poor outcome as scores between 3-6 [18]. Prolonged length of stay (LOS) was defined as hospitalizations >75^th^ percentile of the hospitalization length for the cohort (>6 days) [19, 20]. Finally, TOAST criteria were implemented and each stroke case assigned an appropriate category. Embolism from aortic atheromatous plaques was classified under category 4 (other determined etiology) [21]. Cases with two or more possible etiologies or a negative/incomplete work up were assigned under category 5 (undetermined etiology) [12].

### 2.5. Statistical Analysis

Analysis of the sample demographics and descriptive statistics were done first. The frequency of the prevalence of independent variables (risk factors) was then analyzed. In addition, the frequency of stroke etiology work-up was reported. Chi-square analysis was used to investigate the relationship between categorical risk factors and type of stroke, and univariate analysis of variance “ANOVA” was used to measure the association of continuous risk factors and type of stroke. LOS is represented in graphs and tables using median and interquartile range “IQR” [22], while both the mean and median were used in the text. Logistic regression analysis was then used to measure the contribution of each of the independent variables (including age, sex, NIHSS score on admission, smoking, atrial fibrillation, heart failure, hyperlipidemia, ischemic heart disease, diabetes mellitus, hypertension, and length of hospitalization) to the mRS score at discharge. Lastly, between-group mean difference analysis was done to investigate the relationship between stroke etiology and LOS. All statistical analyses were run in SPSS software. The significance level *α* was set at 0.05.

## 3. Results

### 3.1. Cohort Characteristics

The research team's primary search identified 191 potential cases, of which 142 were included in the final analysis, 13 were excluded due to a better alternative diagnosis, and 36 had a previous stroke.

Of the study population, 76 (53.52%) were males and 66 (46.47%) were females. The mean age for the study population was 66.5 years (SD 12). The age range was 31-94 years. The mean age for males was 66.55 years (SD ± 11.48), and for females was 68.59 years (SD ± 12.53). Stroke in the young was observed in 15 patients (10.56%), of which 10 were males and 5 were females.

### 3.2. Risk Factors

As far as comorbidities are concerned, 112 (78.87%) patients had a previous diagnosis of hypertension, 86 (60.56%) had diabetes mellitus, 53 (37.32%) had hyperlipidemia, 42 (29.47%) had ischemic heart disease, 20 (14.08%) had atrial fibrillation, and 20 (14.08%) had heart failure. Thirty-five (24.64%) of patients were current smokers. See Table 1.

### 3.3. Etiology

Small vessel occlusion was the most commonly identified etiology (54.22%) followed by large artery atherosclerosis (21.83%) and cardioembolic stroke (7.04%). Other causes were determined in 9 cases (6.33%), while 15 cases (10.56%) remain of no definitively determined etiology.

As shown in Table 2, standard stroke work-up consisted of ECG, transthoracic echocardiogram “TTE”, angiography (magnetic resonance angiography “MRA”, Computed tomography angiography “CTA”, and/or Carotid Doppler), and Holter monitoring with transesophageal echocardiogram “TEE” in cases where suspicion of cardioembolism remained high but TTE was inconclusive.

Initial angiography identified 49 patients with significant extra- or intracranial atherosclerotic changes, of whom 31 were ultimately assigned to the large-artery stroke category. Vasculitis was diagnosed in 2 cases. In the remaining patients, the atherosclerosis was either excluded upon definitive testing or corresponded to a different vascular territory. Vascular imaging was not done or suboptimal in 13 cases.

Atrial fibrillation was documented in 20 patients, of whom 10 were ultimately assigned to the cardioembolic category, while the remaining cases were assigned to other categories according to the results of further work up (based on the definitions provided in the methodology section).

The etiologies identified in TOAST category 4 were aortic atherosclerosis (4 cases), vasculitis (3 cases), thrombophilia (1 case), and aortic valve elastochondroma (1 case).

Table 3 illustrates the frequency of each ischemic stroke category, mean age, median LOS, and sex distribution for each group.  Table 4 illustrates the frequency of stroke risk factors for each category.

The left side of the brain was affected by the stroke in 94 cases (66.19%), while strokes affecting the right side of the brain occurred in 46 (32.39%) cases, and 2 cases (1.4%) suffered multiple bilateral strokes.

### 3.4. Severity, Residual Disability, and Their Predictors

Index events resulted in 742 hospitalization days (mean LOS 5.2 days, SD ± 5.1 days; median LOS 4 days, IQR 2.75-6). The mean LOS for males was 5.75 days (SD ± 6.45) and for females was 4.62 (SD ± 3.00). The difference between the two means was statistically significant at *P* < 0.05 (*F* = 6.706, *P* = 0.011).

Prolonged LOS was observed in 33 (23.23%) cases. The commonest cause for prolonged LOS was persistent dysphagia (57.57%), followed by nosocomial infection “NI” (39.39%), or both (21.21%). All patients who suffered from NI had prolonged LOS.  Table 5 illustrates the causes of prolonged LOS and their percentages.

The mean NIHSS score on admission was 7.94 (SD 6.11). Sixty-six (46.15%) patients had a NIHSS score on admission of 5 or less, 57 (40.14%) patients had a score of 5-15, 17 (11.97%) patients had a score of 15-24, while 2 (1.40%) had a score of 25 or more. All patients were offered the same medical care according to the stroke protocol at KAUH. Four patients (2.81%) died in the hospital (immediate mortality), all of whom had a NIHSS score upon admission of 15 or more. Mean discharge NIHSS score for the remaining 138 patients was 5.76 (SD 5.06), of which 86 (60.56%) patients had a score of 5 or less, 44 (30.98%) patients had a score 6-15, 7 (4.92%) patients had a score of 16-25, and 1 (0.70%) had a score higher than 25.

The mean discharge mRS score for the cohort was 2.47 (SD ± 1.79). Patients who are current smokers had the highest mean mRS score compared to patients with medical comorbidities, with a mean of 2.83 (SD ± 1.82). Mean mRS score was, also, compared among the different stroke etiologies (Table 6). Large artery atherosclerosis was associated with the highest residual disability with a mean score of 3.67 (SD ± 1.88), while the stroke of undetermined etiology was associated with the lowest residual disability with a mean score of 1.60 (SD ± 1.78).

To investigate the relationship between the type of stroke and prognosis, Chi-square analysis showed a statistically significant association between different types of stroke and mRS score (*P* 0.007). Table 6 shows that large artery atherosclerosis has the worse disability prognosis, while stroke of undetermined etiology had the best prognosis.

Table 7 shows that even though small vessel occlusion was the most frequent stroke etiology, the severity of stroke was worse in the large-artery etiology group compared with all other types of stroke. The difference between the mean NIHSS score on admission was statistically significant between large artery atherosclerosis and all other types (F14.22, *P* 0.000). The table, also, shows that admission NIHSS score is a significant predictor of mRS score (*t* 9.979, *P* 0.000).

As for risk factors, smokers had a significantly higher admission NIHSS scores than nonsmokers (*t* 3.24, *P* < 0.001). Age, also, was significantly correlated with NIHSS on admission, as patients above the age of 50 were more likely to have a higher mean score of NIHSS admission score compared with younger patients (*t* 1.98, *P* < 0.049). Finally, there was a statistically significant association between admission NIHSS score and the patients' length of stay, with a Pearson correlation coefficient of 0.299, *P* 0.000.

Finally, logistic regression analysis was done to investigate the significant predictors of mRS score. As shown in Table 8, gender was a significant predictor of mRS score, as female patients were more likely to have higher mRS scores than male patients, with an odds ratio of 0.259 (*P* 0.046). Age was, also, a significant predictor of mRS score. Patients above the age of 50 were more likely to have higher mRS score, with an odds ratio of 29.74 (*P* 0.022).

In addition, patients with a stroke due to a small vessel occlusion were more liable than patients with a stroke of undetermined etiology to get mRS score of 3-6 with an odds ratio of 8.335 (*P* 0.45), while patients with a stroke of other determined etiology were more likely to get mRS 3-6 compared to patients with stroke of undetermined etiology by an odds ratio of 2.399. Meanwhile, the other causes of stroke had no significant difference in comparison with each other. NIHSS score on admission was, also, a strong predictor of discharge mRS score, with an odds ratio of 1.99 (*P* 0.000). Other factors were not found to be significant predictors of mRS score.

Table 9 provides a glance at the findings of this study in comparison to the findings of other published local studies.

## 4. Discussion

The findings of this study underscore the central role of hypertension in the pathophysiology of ischemic stroke. The study shows that hypertension was the most prevalent modifiable risk factor (78.87%), both overall and for each TOAST category (except for cardioembolic stroke where it came second to AF). This is consistent with the findings of two previous local studies [8, 9], and with findings of published studies in other countries [6, 23]. The second most common risk factor identified was diabetes mellitus (60.56%), while hyperlipidemia came third, in contrast to the recent survey by Bahou et al., where hyperlipidemia was the second most common. Hypertension (66%) and diabetes mellitus (38%) were also the commonest two risk factors identified in a recent multinational prospective study evaluating stroke characteristics in countries from the Middle East and North Africa “MENA” region compared with patients from non-MENA countries [24].

The results of this study are consistent with previous local studies that showed that lacunar stroke is the most prevalent ischemic stroke etiology in the country. This trend was also noted in recent studies from our region as well as from other regions [25–28]. The prevalence of stroke due to large artery atherosclerosis in our study is also within the range of previous local studies and the global range [8, 9, 29]. The prevalence of cardioembolic stroke (7.04%) was consistent with the previously published local studies which reported an incidence between 6%-8% of stroke etiology. Meanwhile, studies from other regions reported that cardioembolic strokes may account for up to one fifth to one-quarter of ischemic strokes [30–32]. This discrepancy may be a result of poorer control of traditional ischemic stroke risk factors such as hypertension and hyperlipidemia in Jordan in comparison to that in high-income countries. Also, hypertension and diabetes mellitus both have a higher prevalence in Jordan compared with global figures [33, 34]. The discrepancy may, also, reflect the scarcity of resources that compromise the availability or accessibility to investigational techniques, and/or feasibility of patient follow-up in hospitals in low- and middle-income countries. Moreover, differences in methodology may contribute to the noted discrepancy. Some studies list aortic atherosclerosis under the cardioembolic category [35], while the emergence in recent years of the concept of Embolic Stroke of Undetermined Source “ESUS” resulted in a portion of cases being shifted from the undetermined etiology to the cardioembolic category [36]. Another possible reason could be a low level of awareness about the lacunar stroke category as a consequence of uncontrolled hypertension among patients due to either lower educational level or lack of appropriate disease-specific educational programs offered by physicians about hypertension and its complications. Lastly, the retrospective nature of this study could have also contributed to the discrepancy.

The mean age of patients with the first ischemic stroke in Jordan increased over time (Table 9), but it remains younger than the mean age of first stroke reported from high-income countries [37–38]. This is in spite of the population in Jordan being younger [39], which also is attributable—at least in part—to the effects of the limitations in diagnostic, treatment, and prevention modalities in Jordan.

Atrial fibrillation was documented in 20 patients in our study (14%), while the previous local studies in 2004 and 2015 reported atrial fibrillation in 6% and 4% of patients in their cohorts, respectively. This could be partially related to the greater commercial and logistical availability of ambulatory cardiac monitors nowadays. Also, the medical registry at KAUH is completely electronic, which offered the advantage of retrieving previously documented paroxysmal atrial fibrillation in some patients who presented with acute stroke while having a sinus rhythm. Furthermore, our findings are in line with other recent studies in the region. For instance, atrial fibrillation was found in 13% of the patients in a Saudi study published in 2016 [28], while older studies from the same region reported lower numbers (5% and 10%) [40, 41]. Upon reviewing the dates of the referenced studies, the reader can observe the temporal trend of increasing detection of atrial fibrillation in stroke cohorts in the region.

On the other hand, smoking was documented in 24.6% of patients in our cohort, while the previous studies from Jordan reported a higher prevalence (35% and 40%). This may be in part due to the interim introduction of new tobacco taxes over the past decade [42]. Also, smoking is more prevalent among Jordanian males, and the male to female ratio in our study was lower than the other studies [42]. Further, no clear trends are delineated when smoking statistics between the years 2000-2016 are compared, but the overall reported percentages of daily smokers had a wide range between 24-32% among Jordanians [42].

Twenty (14.08%) of the patients in our cohort developed NI, mainly pneumonia (8 patients, 5.63%), UTI (8 patients, 5.63%), and both (2 patients, 1.40%). Studies in other regions that sought to determine the rate of NI after acute ischemic stroke reported a wide range (6-48.3%), with pneumonia and UTI being the most frequently encountered NIs [43–48], while studies evaluating NI after strokes due to ischemic and hemorrhagic etiologies combined reported an even higher incidence (up to 65%) [49–52]. Meanwhile, a retrospective cohort study conducted at KAUH in 2019 which evaluated NI in patients with intracranial hemorrhage found the incidence of NI to be 40.3% overall and 46% among patients with intracerebral hemorrhage “ICH” [53]. The incidence of NIs in our study falls within the range that has been reported in other published studies.

Age, smoking, and large-artery etiology were significantly associated with higher stroke severity on admission. Age was also a significant predictor of a higher residual disability, in addition to female sex and stroke severity on admission. These relations have been reported in previous studies [54–59]. Logistic regression also demonstrated that lacunar strokes and strokes due to other determined etiologies predicted worse outcomes when compared with strokes due to undetermined etiology, but similar associations could not be drawn upon comparing the other etiologies. On the other hand, our analysis demonstrated an association between large-artery etiology and residual disability, and as mentioned above, the large-artery etiology was also associated with higher severity on presentation. Together, these findings point indirectly towards an association between large artery atherosclerosis and unfavorable stroke outcome.

The strengths of this study include the rigorous case ascertainment and data collection process, which allowed for a deeper insight into the epidemiology and prevalence of risk factors among patients with a first ischemic stroke in Jordan. It is also the first study to shed light on stroke severity, stroke-related disability, and its predictors among Jordanian patients, which can be viewed as a platform to launch larger and prospective future studies. At the same time, this study has several limitations. It is a single-center study that included patients from KAUH only, which has led to a relatively small sample, and consequently limited the type of statistical analysis techniques that could have been used. Also, it was a pure retrospective study, where patient follow-up was not feasible. Finally, cardioembolic work-up could have been more comprehensive. Future studies could include a more complete and rigorous evaluation of possible cardiovascular etiologies by having cardiologists as a part of the investigation team.

## 5. Conclusion

Ischemic strokes have different etiologies that are associated with different levels of impact on the patient's clinical status and prognosis. Hypertension is the most common comorbidity found in patients admitted for stroke. Small-vessel occlusion was the most frequent stroke etiology. However, large artery atherosclerosis was associated with the highest residual disability. Regarding predictors of prognosis, current smoking status, and age above 50 were and NIHSS on admission appear to be the strongest predictors of prognosis. Finally, higher NIHSS score on admission resulted in a longer hospital stay.

## Figures and Tables

**Table 1:** Stroke risk factors and their distribution by sex. Abbreviations: ischemic heart disease: IHD.

Risk factor	Number (percentage)	Gender	
		Male	Female
Hypertension	112 (78.87%)	55 (72.36%)	57 (86.36%)
Diabetes mellitus	86 (60.56%)	45 (59.21%)	41 (62.12%)
IHD	42 (29.47%)	32 (42.1%)	10 (15.15%)
Hyperlipidemia	53 (37.32%)	29 (38.15%)	24 (36.36%)
Smoking	35 (24.64%)	29 (38.15%)	6 (9.09%)
Atrial fibrillation	20 (14.08%)	7 (9.21%)	13 (19.69%)
Heart failure	20 (14.08%)	10 (13.15%)	10 (15.15%)

**Table 2:** Frequency of procedures performed under stroke work-up (Note: numbers are not mutually exclusive).

Type of procedure	Number performed	Percentage of patients
Electrocardiogram (ECG)	141	99.29%
Transthoracic Echo (TTE)	138	97.18%
Holter and transesophageal Echo (TEE)	16	11.26%
MRA	59	41.54%
CTA	72	50.70%
Carotid Doppler	11	7.74%
4-vessel angiography	1	0.70%

**Table 3:** Stroke categories and their frequency by sex.

TOAST category	Number (%)	Mean age (SD)	Mean LOS (SD)	Median LOS (days)	Sex	
					Male (%)	Female (%)
Large-artery atherosclerosis	31 (21.83%)	70.7 (10.2)	7.61 (±5.84)	6	16 (21.05%)	15 (22.72%)
Cardioembolism	10 (7.04%)	72.5 (7.91)	4.20 (±1.47)	4	4 (5.26%)	6 (9.09%)
Small-vessel occlusion	77 (54.22%)	66.03 (10.84)	4.72 (±5.42)	3	47 (61.84%)	30 (45.45%)
Other determined etiology	9 (6.33%)	52.33 (16.68)	5.55 (±3.81)	4	4 (5.26%)	5 (7.57%)
Undetermined etiology	15 (10.56%)	65.13 (12.67)	3.33 (±2.66)	2	5 (6.57%)	10 (15.15%)

**Table 4:** Prevalence (*n*) and percentage (%) of stroke risk factors for each stroke category. Abbreviations: Ischemic heart disease: IHD; atrial fibrillation: AF; heart failure: HF.

TOAST category	Hypertension	Diabetes	IHD	Hyperlipidemia	Smoking	AF	HF
Large-artery atherosclerosis	27 (87.09%)	19 (61.29%)	7 (22.58%)	12 (38.7%)	11 (35.48%)	6 (19.35%)	6 (19.35%)
Cardioembolism	8 (80%)	5 (50%)	3 (30%)	2 (20%)	2 (20%)	10 (100%)	5 (50%)
Small-vessel occlusion	60 (77.92%)	50 (64.93%)	25 (32.46%)	31 (40.25%)	17 (22.07%)	2 (2.59%)	7 (9.09%)
Other determined etiology	4 (44.44%)	3 (33.33%)	3 (33.33%)	3 (33.33%)	2 (22.22%)	0 (0%)	0 (0%)
Undetermined etiology	13 (86.67%)	9 (60%)	4 (26.67%)	5 (33.33%)	3 (20%)	2 (13.33%)	2 (13.33%)

**Table 5:** Reasons for prolonged LOS. Abbreviations: urinary tract infection: UTI; Percutaneous endoscopic gastrostomy: PEG. Note: classification is not mutually exclusive.

Reason for prolonged LOS	
Nosocomial infection	13 (39.39%)
Pneumonia	5 (38.46%)
UTI	4 (30.76%)
Both pneumonia and UTI	2 (15.38%)
Other infections	2 (15.38%)
Dysphagia	19 (57.57%)
Dysphagia requiring PEG tube	12 (63.15%)
Dysphagia resolving during admission	7 (36.85%)
Both dysphagia and nosocomial infection	7 (21.21%)
Pneumonia	3 (42.85%)
UTI	4 (57.14%)
Both pneumonia and UTI	0 (0%)
Other infections	0 (0%)
Dysphagia requiring PEG tube	7 (100%%)
Dysphagia resolving during admission	0 (0%)
Others	
Decreased consciousness	2 (6.06%)
Optimization of anticoagulation	3 (9.09%)
Hemorrhagic transformation	2 (6.06%)
Observation for other complications	5 (15.15%)
Pending further investigations	3 (9.09%)

**Table 6:** Mean mRS score for each stroke etiology and the correlation between stroke etiology and mRS scores.

TOAST category		Mean mRS		Number		Standard deviation	
Large-artery atherosclerosis		3.6774		31		1.88657	
Cardioembolism		2.6000		10		1.64655	
Small-vessel occlusion		2.2078		77		1.58395	
Other determined etiology		1.8889		9		1.69148	
Undetermined etiology		1.6000		15		1.72378	
All categories		2.4718		142		1.78923	

Chi-square							
mRS score		Type of stroke					Sig: 0.007
		1	2	3	4	5	Total
0-2	*N*	7	5	42	6	11	71
	%	22.6	50	54.5	66.7	73.3	
3-6	*N*	24	5	35	3	4	71
	%	77.4	50	45.5	33.3	26.7	

**Table 7:** Variables with statistically significant correlation with the admission NIHSS score.

Variables	*N*	Mean NIHSS admission	Standard deviation	Factor	Value	Sig
Type of stroke						
Large artery atherosclerosis	31	14.098	7.95133	F	14.228	0.000
Cardioembolism	10	8.4000	6.97199			
Small vessel occlusion	77	5.9870	3.59639			
Stroke of other determined etiology	9	5.4444	4.45724			
Stroke of undetermined etiology	15	6.4667	4.68838			
Smoking						
Yes	35	10.7714	7.55406	T	3.244	0.001
No	107	7.0187	5.32169			
mRS score						
0-2	71	4	2.23607	T	9.979	0.000
3-6	71	11.8873	6.27364			
Age						
Less than 50 years old	15	5.000	4.69586	T	1.984	0.049
More than 50 years old	127	8.2913	6.21329			

**Table 8:** Logistic regression on the impact of independent variables on mRS score.

Variables	*B*	Wald statistic	Significance	Odds
Gender	-1.352	3.965	0.046	0.259
Age (reference less than 50)	3.393	5.216	0.022	29.746
Smoking	0.180	0.057	0.811	1.197
Dyslipidemia	-0.046	0.006	0.940	0.955
Atrial fibrillation	0.100	0.005	0.945	1.105
Diabetes	0.582	0.841	0.359	1.790
Hypertension	-0.055	0.005	0.946	0.946
Ischemic heart disease	-0.876	1.507	0.220	0.416
TOAST-subtype		5.515	0.238	
(1) Large artery atherosclerosis	1.736	1.524	.217	5.674
(2) Cardioembolism	0.672	0.117	.733	1.958
(3) Small-vessel occlusion	2.120	4.037	.045	8.335
(4) Stroke of other determined etiology	3.346	3.908	.048	2.399
NIHSS-admission	0.645	25.021	0.000	1.907
Length of stay per days	0.074	1.253	0.263	1.077
Heart failure	0.945	0.985	0.321	2.572

**Table 9:** Prevalence of risk factors, mean age, male prevalence, and stroke categories in 3 local studies from Jordan.

	Our data (2017-2018)	Bahou et al., 2015	Bahou et al., 2004
Risk factors			
Hypertension	78.87%	85%	76%
Diabetes mellitus	60.56%	65%	44%
IHD	29.47%	39%	20.5%
Hyperlipidemia	37.32%	71%	33%
Smoking	24.64%	40%	35%
Atrial fibrillation	14.08%	4%	6%
Heart failure	14.08%	Not reported	Not reported
Male prevalence (%)	53.52%	62%	56%
Mean age (years)	66.5	66	61.2
TOAST category			
Large-artery atherosclerosis	21.83%	28%	20%
Cardioembolism	7.04%	6%	8%
Small-vessel occlusion	54.22%	36%	51.5%
Other determined etiology	6.33%	Not reported	Not reported
Undetermined etiology	10.56%	30%	19.5%

## Data Availability

Study data is available upon reasonable request.
